# Elimination of Decapod iridovirus 1 (DIV1) infection at high water temperature: a new environmental control strategy

**DOI:** 10.1007/s44307-024-00012-0

**Published:** 2024-03-18

**Authors:** Yue Wang, Linxin Dai, Zuluan Liang, Naijie Hu, Danqing Hou, Yinhuan Zhou, Chengbo Sun

**Affiliations:** 1 College of Fisheries, Guangdong Ocean University, Zhanjiang, Guangdong China; 2 Guangdong Provincial Laboratory of Southern Marine Science and Engineering, Zhanjiang, Guangdong China; 3 Guangdong Provincial Key Laboratory of Pathogenic Biology and Epidemiology for Aquatic Economic Animals, Zhanjiang, Guangdong China

**Keywords:** Decapod iridovirus 1 (DIV1), *Penaeus monodon*, Water temperature, Change temperature, Latent infection, Acute infection

## Abstract

Decapod iridovirus 1 (DIV1) poses a major challenge to sustainable shrimp farming and poses a serious hazard to aquaculture industry. This study investigated the complex interaction between DIV1 infection and water temperature, focusing on the effect of high temperature on DIV1 infection due to *Penaeus monodon*. Using models of latent and acute infection, the study revealed the response of *P. monodon* to DIV1 under different conditions. In the experimental set-up, the effect of high water temperature (34 ± 1 °C) compared with room temperature (26 ± 1 °C) was investigated. DIV1 replication was significantly inhibited in the high-temperature group (H), resulting in complete viral elimination within 15 days. DIV1 did not resurface even after return to room temperature (26 ± 1 °C), indicating sustained antiviral effects. Compared with the room temperature (26 ± 1 °C) group (N), the H group showed a 100% reduction in the incidence of latent and acute infection. Exposure to high water temperature directly impaired the viability of DIV1, enhancing the immune system of *P. monodon*, and expediting metabolic processes for efficient DIV1 clearance. The study highlights the significant inhibitory effects of high water temperature (34 ± 1 °C) on DIV1 infection in *P. monodon,* resulting in viral eradication. This discovery offers a potential strategy for mitigating DIV1 infections in shrimp aquaculture, prompting further investigation into underlying mechanisms. Optimising parameters and protocols for high-temperature treatment is crucial for viral control. Exploring the broader implications of the findings on other viral infections in crustacean aquaculture could provide valuable insights for comprehensive disease prevention and control.

## Introduction

Shrimp, an arthropod commonly found in freshwater and marine ecosystems, represents a very popular aquatic species, not only as a delicacy but also for its high nutritional and health value (Abdel-Latif et al. 2022; Hicks et al. 2019; Jihongwu 2010). Shrimps are categorized into many different species, including *P. monodon,* which is widely distributed in the coastal areas of Asia and Africa. *P. monodon* has a high economic value and is one of the most important export commodities for many coastal countries. In aquaculture, *P. monodon* is widely reared (Viet Nguyen et al. 2020). Effective aquaculture management of *P. monodon* can lead to substantial economic benefits for farmers.

However, the farming of *P. monodon* is also associated with challenges. One of the most serious challenges is the outbreak and spread of diseases. Due to the weak immune system of *P. monodon* during growth, it is susceptible to a variety of pathogens that can reduce the farming efficiency and lead to economic losses.

DIV1 is one of the most common pathogens associated with shrimp. Infection with DIV1 can lead to severe lesions, growth and even death of shrimp. DIV1 is a large, icosahedral, cytoplasmic virus containing a linear double-stranded DNA with a diameter of 120–300 nm and a core size of 80–90 nm (Marschang 2011; Paperna et al. 2001). It is an iridescent virus because of its bluish-purple coloration when exposed to oblique light (Zhang, 2014). DIV1 has a wide range of hosts, primarily invertebrates and metazoan vertebrates (Chinchar 2002; Chinchar et al. 2011; Williams 2008; Williams et al. 2005). The iridoviridae were first discovered in 1954 in the British region from *Tiphoneula paludosa* and named as Marsh Aedes iridovirus (XEROS 1954). Since then, members of the family iridoviridae have been detected in crustaceans, fish, reptiles, and amphibians (Chinchar et al. 2011; Matthews 1979; Xu et al. 2016). In 2007, Tang K F et al. (Tang et al. 2007) isolated a strain of iridovirus from *Aceteserythraeus* belonging to family Sakuraidae and designated it as Sergestid iridovirus (SIV) SIV infection resulted in massive death of shrimp. In 2016, a novel iridovirus was detected in a *Cherax quadricarinatus* farm in Fujian Province, China, and named Cherax quadricarinatus iridovirus (CQIV) *C. quadricarinatus* is a major host for CQIV. CQIV has a very high lethality rate in *Procambarus clarkii* and *Litopenaeus vannamei* (Xu et al. 2016). In 2017, Qiu L et al. (Qiu et al. 2017) identified an extremely lethal strain of iridovirus named Shrimp hemocyte iridescent virus (SHIV). In March 2019, the Executive Committee of the International Committee on Taxonomy of Viruses (ICTV) identified SHIV 20141215 and CQIV CN01 as two viral isolates of Decapod iridescent virus1 (DIV1) (Baker et al. 2000; Williams 1994). To date, *L. vannamei* (Qiu et al. 2017, 2018), *P. monodon* (He et al. 2021; Srisala et al. 2021), *Fenneropenaeus chinensis* (Liao et al. 2020), *Marsupenaeus japonicus* (He et al. 2022a, b; He et al. 2022a, b), *Metapenaeus ensis* (Liao et al. 2022), *C. quadricarinatus* (Qiu et al. 2018; Xu et al. 2016), *Macrobrachium rosenbergii* (Qiu et al. 2019), *Macrobrachium nipponense* (Qiu et al. 2019), *Pro. clarkii* (Qiu et al. 2019), *Exopalaemon carinicauda* (Chen et al. 2019), *Portunus trituberculatus*, and *Pachygrapsus crassipes* (Chen, 2019) have been identified as hosts of DIV1 (Wang et al. 2023).

In a recent study, He (He et al. 2021) modeled latent and acute infections and determined the thresholds for latent and acute infections by investigating DIV1 infection in *P. monodon*. According to the study, when the injected concentration of DIV1 was ≤ 1.15 × 10^6^ copies/μg DNA, the zebra shrimp exhibited latent infection. No viral outbreak was apparent at that concentration. The shrimp showed no clinical symptoms or lethal effects. However, when the injected concentration of DIV1 was ≥ 1.15 × 10^7^ copies/μg DNA, the prawns entered a state of acute infection, which resulted in viral outbreak and death of the prawns. He's study revealed two stages of DIV1 infection in *P. monodon*, namely latent and acute stages. During latent infection, the virus continuously infects the host. In this case, the virion is present in some tissues of the host, but not at levels enough to trigger an outbreak of disease, so there are no clinical symptoms or death. However, when the environmental factors are altered, such as stress, the shrimps’ immunity decreases, which leads to the transformation from a state of latent infection to a state of acute infection, resulting in viral outbreak and the death of shrimps eventually.

The host-virus-environment shows a delicate and dynamic balance. Water temperature plays an important role in the growth, development, reproduction, metabolism and immunity of *P. monodon* (Abdelrahman et al. 2019; Wang et al. 2020; Wyban et al. 1995; Zhou et al. 2010). Generally, the immune system and disease resistance of *P. monodon* are stronger at ambient water temperature. However, the immune system of *P. monodon* is significantly reduced under excessively high or low temperatures, thus increasing the susceptibility to infections by various pathogens. Interestingly, however, studies have shown that increasing water temperature can partially suppress and eliminate viruses (Du et al. 2008; Du et al. 2006; Granja et al. 2006; Guo et al. 2022; Jiravanichpaisal et al. 2004; Rahman et al. 2006; Withyachumnarnkul et al. 2003; You et al. 2010; Luo, 2010; Yuan, 2020).

In this study, we first developed a model of DIV1 infection (latent and acute) involving *P. monodon*. The shrimps were exposed to high water temperature (34 ± 1 °C), which eliminated DIV1 infection. The findings were validated to ensure that the virus does not resurrect in high-temperature-treated *P. monodon* following return to room temperature (26 ± 1 °C). This experiment is significant because it not only reveals the mechanism of DIV1 infection in *P. monodon*, but also provides new insights into mechanisms of viral resistance in crustaceans. In addition, the results provide a brand new strategy for breeding and culture of specific pathogen free (SPF) *P. monodon* species. Overall, this study provides a strong support for our understanding of DIV1 infection and immune mechanisms in *P. monodon*, and provides new ideas for further investigation.

## Materials and methods

### Animals

Healthy *P. monodon* individuals were obtained from pool 13 of 12 m^3^ in workshop C of the Donghai Island Marine Biology Research Base of Guangdong Ocean University, and were temporarily cultured for DIV1 infection in vitro. The average body length of the shrimp was 10.2 ± 0.3 cm, and the average body mass was 14.65 ± 0.2 g. After 7 days of culture in pool 13, the shrimps were randomly assigned to the negative control group, the DIV1-infected group at 26 ± 1 °C, and the DIV1-infected group at 34 ± 1 °C. Each 60 L bucket carried 10 shrimps. A total of 30 shrimps were cultured in each set of three parallels. The cultured *P. monodon* species were fed with artificial feed twice a day. The water was changed completely once a day. The water temperature during the experimental period was 26 ± 1 °C. The pH was 7.9 ± 0.5. The salinity was 27‰. Five shrimps were randomly selected for PCR testing for DIV1, WSSV, IHHNV, and *Vibrio anisopliae* prior to experimentation, and the test results were all negative.

### Preparation of DIV1 inoculum

Intramuscular injection was used to infect five healthy *P. monodon* individuals cultured in pool 13 to revive the virus. Following the injection, the shrimps were monitored. Muscle tissues of dying *P. monodon* were stored at -80 °C until DNA was extracted. The infected *P. monodon* individuals were subjected to nested PCR to test for DIV1 using the assay method of Qiu (Chen et al. 2019) et al. *P. monodon* that tested positive were analysed by the probe method. The muscle viral load was measured via qRT-PCR as described by Qiu (Chen et al. 2019) et al. The primers used for DIV1 detection and quantification are shown in Table 1. *P. monodon* with high muscle virus carriage were selected for the preparation of DIV1 inoculum, and 0.3 g of shelled muscle tissue was cut into sterile 1.5 mL EP tubes and minced. Following addition of 1 mL of pre-cooled sterile high-salt phosphate-buffered saline (PBS, pH 7.4), the solution was centrifuged at 4 °C and 10,000 rpm for 10 min. The supernatant was filtered and sterilised with a polycarbonate filter membrane with a pore size of 0.45 μm to obtain the DIV1 virus inoculum. The DIV1 crude extract was diluted with PBS along a tenfold gradient, and seven serial dilution gradients (1.12 × 10^2^, 1.12 × 10^3^, 1.12 × 10^4^, 1.12 × 10^5^, 1.12 × 10^6^, 1.12 × 10^7^, and 1.12 × 10^8^ copies/μg of DNA) were selected for the virulence assay.

**Table 1:** PCR and qPCR primers for the detection of DIV1

No	Primer names	Sequences(5′-3′)
Nested PCR		
1	F1-DIV1	GGGCGGGAGATGGTGTTAGAT
2	R1-DIV1	TCGTTTCGGTACGAAGATGTA
3	F2-DIV1	CGGGAAACGATTCGTATTGGG
4	R2-DIV1	TTGCTTGATCGGCATCCTTGA
qPCR		
7	F-qRT-DIV1	TCGTTTCGGTACGAAGATGTA
8	R-qRT-DIV1	TTTCACACTTCCTGATAGTCTTCCAT
9	DIV1 Taqman Probe	TCACAGAAAAGATTCCCGAAATGGTAAAAC

### Toxicity testing

The experiments were conducted in three parallel groups, with 10 healthy shrimps each. The infection was carried out by intramuscular injection. The virus group was injected with 50 μL of DIV1 crude extract between the 2nd and 3rd abdominal muscles toward the heart, while the control group was injected with 50 μL of PBS buffer at the same position. The death of shrimps was observed and recorded every 4 h, and the dying shrimps were removed promptly to avoid secondary infection, until the death of shrimp was stabilised. During the experiment, the shrimp were fed artificial bait twice daily. The water was changed almost 100% once daily.

### Culture water temperature and group settings

**Fig. 1:**
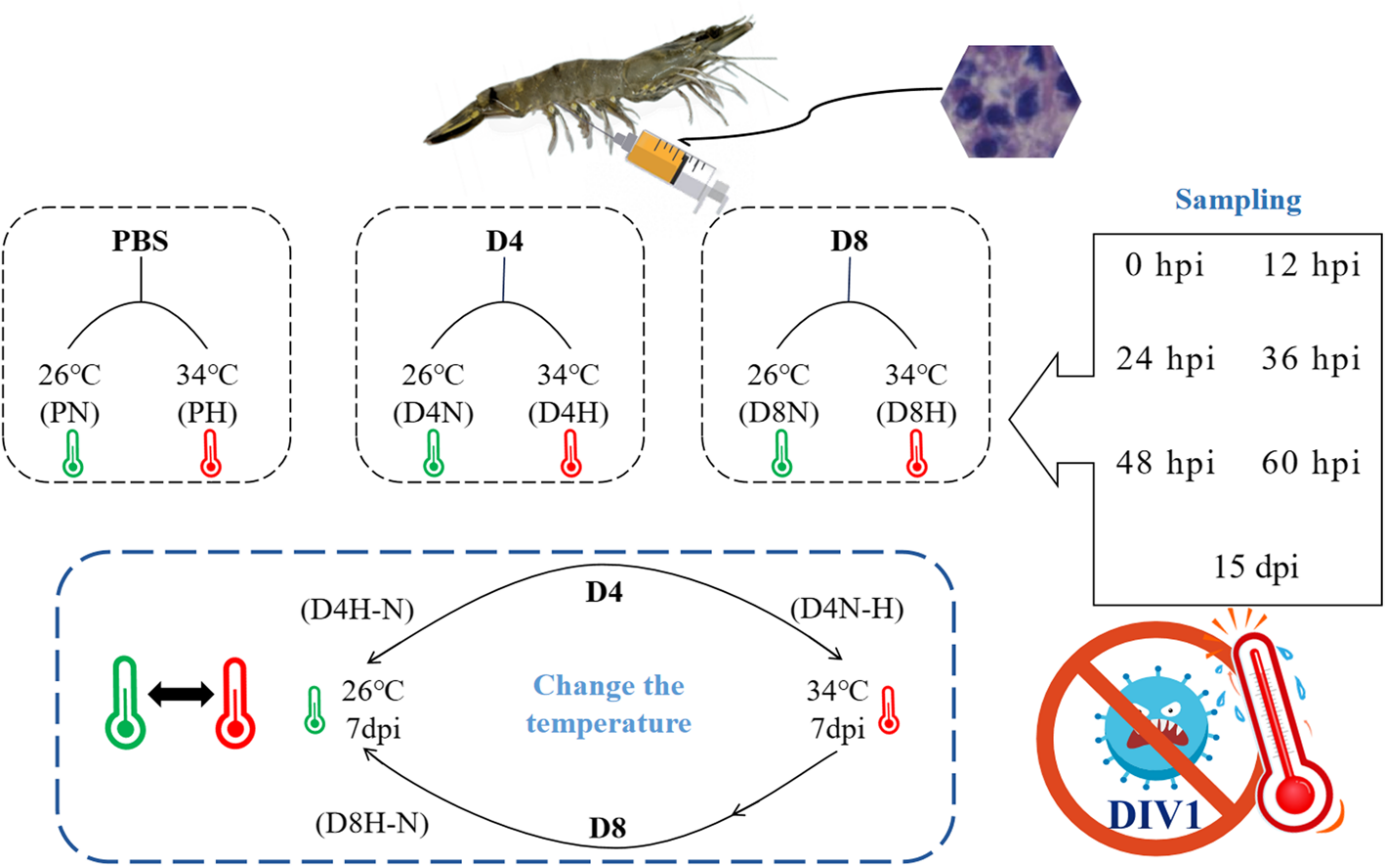
Experimental water temperature and group settings. The group names are indicated in parentheses. PN: Negative control group at room temperature (26 ± 1 °C). PH: Negative control group at high temperature (34 ± 1 °C). D4N: *P. monodon* injected with 1.12 × 10^4^ copies/μg DNA at 26 ± 1 °C. D4H: *P. monodon* injected with 1.12 × 10^4^ copies/μg DNA at 34 ± 1 °C. D8N: *P. monodon* injected with 1.12 × 10^8^ copies/μg DNA at 26 ± 1 °C. D8H: *P. monodon* injected with 1.12 × 10^8^ copies/μg DNA at 34 ± 1 °C. D4N-H: Variable temperature; started at 26 ± 1 °C for 7 days, then transferred to 34 ± 1 °C for another 7 days. D4H-N: Variable temperature; started at 34 ± 1 °C for 7 days, then transferred to 26 ± 1 °C for another 7 days. D8N-H: Variable temperature; started at 34 ± 1 °C for 7 days, then transferred to 26 ± 1 °C for another 7 days

The experimental variables included two different temperatures, two viral gradients and a negative control: 26 ± 1 °C (normothermic group, N), 34 ± 1 °C (hyperthermic group, H), 1.12 × 10^4^ copies/μg DNA (latently infected group, D4), 1.12 × 10^8^ copies/μg DNA (acutely infected group, D8), and a negative control (PBS). The specific groups are shown in Fig. 1. The negative control group (PN) included shrimps in water at room temperature (26 ± 1 °C) and a negative control group (PH) in which the water temperature was high (34 ± 1 °C). The second group consisted of *P. monodon* injected with 1.12 × 10^4^ copies/μg DNA at a water temperature of 26 ± 1 °C for rearing (D4N) and at 34 ± 1 °C for rearing (D4H). In the third group, *P. monodon* injected with 1.12 × 10^8^ copies/μg DNA were placed at water temperature 26 ± 1 °C for rearing (D8N) and at 34 ± 1 °C for rearing (D8H). In addition, experiments at variable temperature were set up in which *P. monodon* injected with 1.12 × 10^4^ copies/μg of DNA were placed at a water temperature of 26 ± 1 °C for 7 days and then transferred to a high water temperature of 34 ± 1 °C for another 7 days (D4N-H), a high water temperature of 34 ± 1 °C for 7 days and then transferred to a water temperature of 26 ± 1 °C for another 7 days (D4H- N). *P. monodon* injected with 1.12 × 10^8^ copies/μg DNA were reared at a high water temperature of 34 ± 1 °C for 7 days and then transferred to a water temperature of 26 ± 1 °C for 7 days (D8N-H).

### Sampling

Hepatopancreas, hemolymphocytes, and muscles of *P. monodon* were extracted at 0h, 12 h, 24 h, 12 h, 36 h, 48 h, and 60 h post-injection (hpi) after injection, and at 15 days post-injection (dpi). DNA was extracted for PCR and qPCR to determine the viral copy number. In addition to obtaining hepatopancreas, hemolymphocytes, and muscle on day 7 of culture at each temperature and extracting DNA for PCR and qPCR, the muscles and hepatopancreas obtained from the group at variable temperatures were analysed via transmission electron microscopy. Samples for PCR and qPCR assays were conducted in triplicate in each group, transferred to 1.5 mL EP tubes, and rapidly refrigerated at -80 °C for storage until assay.

### Quantitative analysis of DIV1 using nested PCR and TaqMan qPCR

Total DNA was extracted from hepatopancreas, hemolymphocytes, and muscle tissue samples stored at -80 °C using the *EasyPure*® Marine Animal Genomic DNA Kit (TransGen Biotech,China) according to the instructions. The DNA concentration and purity were determined using the SimpliNano Nucleic Acid Concentration Analyzer (GE Healthcare, USA) to determine the DNA concentration and purity.

The total system was adjusted to 20 μL using 1 μL of extracted DNA. The extracted DNA was then analysed with a CFX96 Real-Time PCR Detection System real-time fluorescence quantitative PCR instrument (Bio-Rad USA) via TaqMan qPCR amplification. The PCR conditions were as follows: 95 °C for 30 s; 95 °C for 5 s; and 60 °C for 30 s, with 40 cycles. The viral copy number of DIV1 in different tissues was calculated, based on the results. The qPCR-specific primers and TaqMan probes used are shown in Table 1.

### Histopathological testing

Intestinal samples were obtained from *P. monodon* using 10% formalin for 24 h at 4 °C and then dehydrated using different concentrations of ethanol. The tissues were made transparent using xylene and embedded in quasi-wax. The tissues were cut to 5 mm thickness and collected on clean slides for hematoxylin and eosin (H&E) staining. The images of the intestinal sections were analysed and recorded using an intestinal section microscope (Olympus, Nikon, Tokyo, Japan) to determine the effects of DIV1 invasion on the intestinal tract of the shrimp.

### Statistical analysis

Data are expressed as mean ± standard deviation ( SD). Data normality was tested using the Shapiro–Wilk method. The data were analysed using SPSS 19.0 ( SPSS Inc., Chicago, IL, USA) and one-way analysis of variance ( ANOVA). Tukey’s multiple comparisons test was used to compare significant differences. Differences were considered statistically significant at P < 0.05. Differences between groups were analysed via Mantel-Cox ( log-rank χ 2 test) method using Graph Pad Prism software ( v8.0.1). The probability unit method in the SPSS 19.0 program ( SPSS Inc., Chicago, IL, USA) was used for probit analysis to calculate LC_50_.

## Results

### Survival of *P. monodon* at different concentrations of DIV1 and different temperatures

**Fig. 2:**
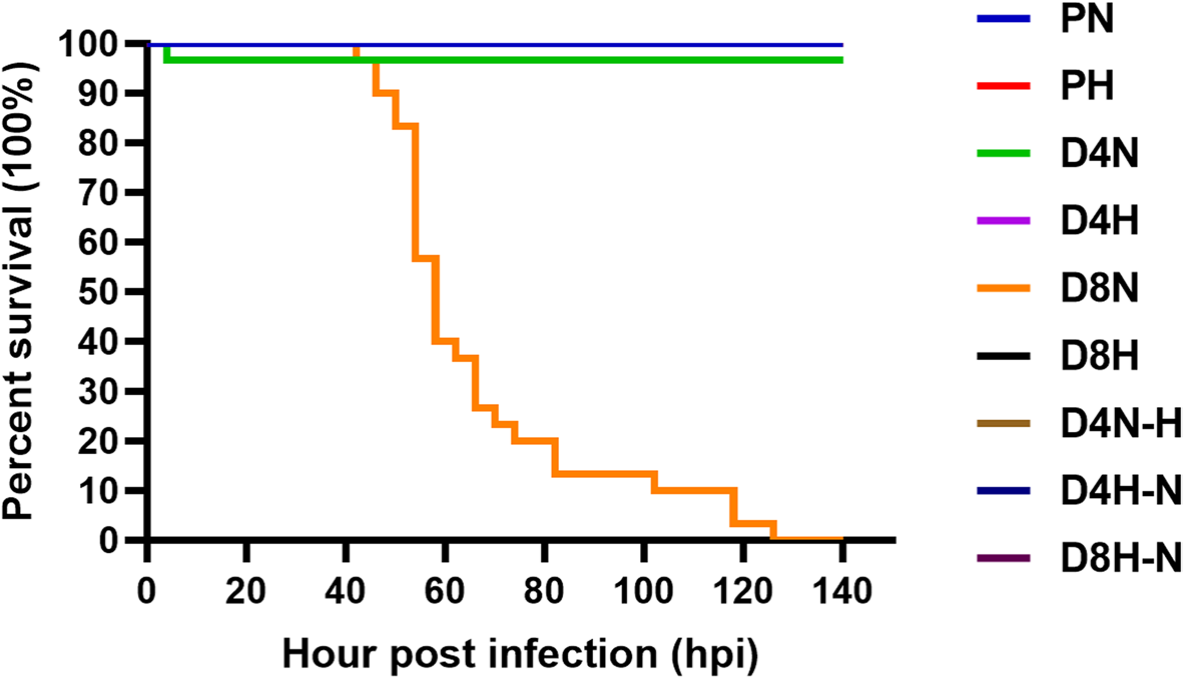
Survival curves of *P. monodon* at different concentrations and temperatures. PN (26 ± 1 °C): PBS negative control, 26 ± 1 °C water temperature. PH (34 ± 1 °C): PBS negative control, 34 ± 1 °C high water temperature. D4N (26 ± 1 °C): DIV1 injected at a concentration of 1.12 × 10^4^ copies/μg DNA, 26 ± 1 °C water temperature. D4H (34 ± 1 °C): DIV1 injected at a concentration of 1.12 × 10^4^ copies/μg DNA, 34 ± 1 °C water temperature. D8N (26 ± 1 °C): DIV1 injected at a concentration of 1.12 × 10^8^ copies/μg DNA, 26 ± 1 °C water temperature. D8H (34 ± 1 °C): DIV1 injected at a concentration of1.12 × 10^8^ copies/μg DNA, 34 ± 1 °C water temperature. D4N-H: transferred to 34 ± 1 °C high water temperature after 7 days on D4N. D4H-N: transferred to 26 ± 1 °C water temperature after 7 days on D4H. D8H-N: transferred to 26 ± 1 °C water temperature after 7 days on D8H. The survival curves of PH、D4H、D8H、D4N-H、D4H-N and D8H-N show a phenomenon of overlapping curves with PN in Figure

The mortality of *P. monodon* after injection of different concentrations of DIV1 viral fluids into the body is shown in Fig. 2. *P. monodon* (PN, PH) in the control group did not die when cultured in both room and high water temperatures (26 ± 1 °C and 34 ± 1 °C, respectively). *P. monodon* in latent infection stage also did not die either at room temperature (26 ± 1 °C) or high water temperature (34 ± 1 °C), as shown in the figure (D4N, D4H). One case of accidental death at 4 hpi in D4N group was analysed to exclude viral lethality, which may be due to accidental death after artificial injection. Therefore, individual cases were excluded from the data. *P. monodon* individuals at acute stage of infection, D8N were cultured in room temperature water (26 ± 1 °C). The results showed that the number of deaths increased with time until 128 hpi, when the mortality rate reached 100%. However, none of the individuals in D8H cultured at high water temperatures (34 ± 1 °C) died. In the variable temperature test groups (D4N-H, D4H-N, and D8H-N), no mortality in any *P. monodon* individuals was detected. The survival curves of PH、D4H、D8H、D4N-H、D4H-N and D8H-N show a phenomenon of overlapping curves with PN in Fig. 2. The LC_50_ values of at 48 hpi, 72 hpi, 96 hpi and 132 hpi were calculated using the probabilistic unit method, as shown in Table 2, which were 2.60 × 10^8^, 8.30 × 10^7^, 6.65 × 10^7^ and 8.79 × 10^6^ copies/μg DNA, respectively. The 95% confidence intervals of the LC_50_ values were 9.29 × 10^7^ ~ 1.31 × 10^9^, 3.36 × 10^7^ ~ 2.59 × 10^8^, 2.94 × 10^7^ ~ 2.18 × 10^8^ and 1.42 × 10^6^ ~ 1.91 × 10^7^ copies/μg DNA, respectively.

**Table 2:** LC_50_ and 95% confidence intervals for DIV1 virus infection of *P. monodon* at normal temperature

Time (hpi)	48	72	96	132
LC_50_ (copies/μg DNA)	2.60 × 10^8^	8.30 × 10^7^	6.65 × 10^7^	8.79 × 10^6^
95% confidence intervals	9.29 × 10^7^ ~ 1.31 × 10^9^	3.36 × 10^7^ ~ 2.59 × 10^8^	2.94 × 10^7^ ~ 2.18 × 10^8^	1.42 × 10^6^ ~ 1.91 × 10^7^

### Clinical symptoms and Histopathological analysis of DIV1 infection in *P. monodon*

**Fig. 3:**
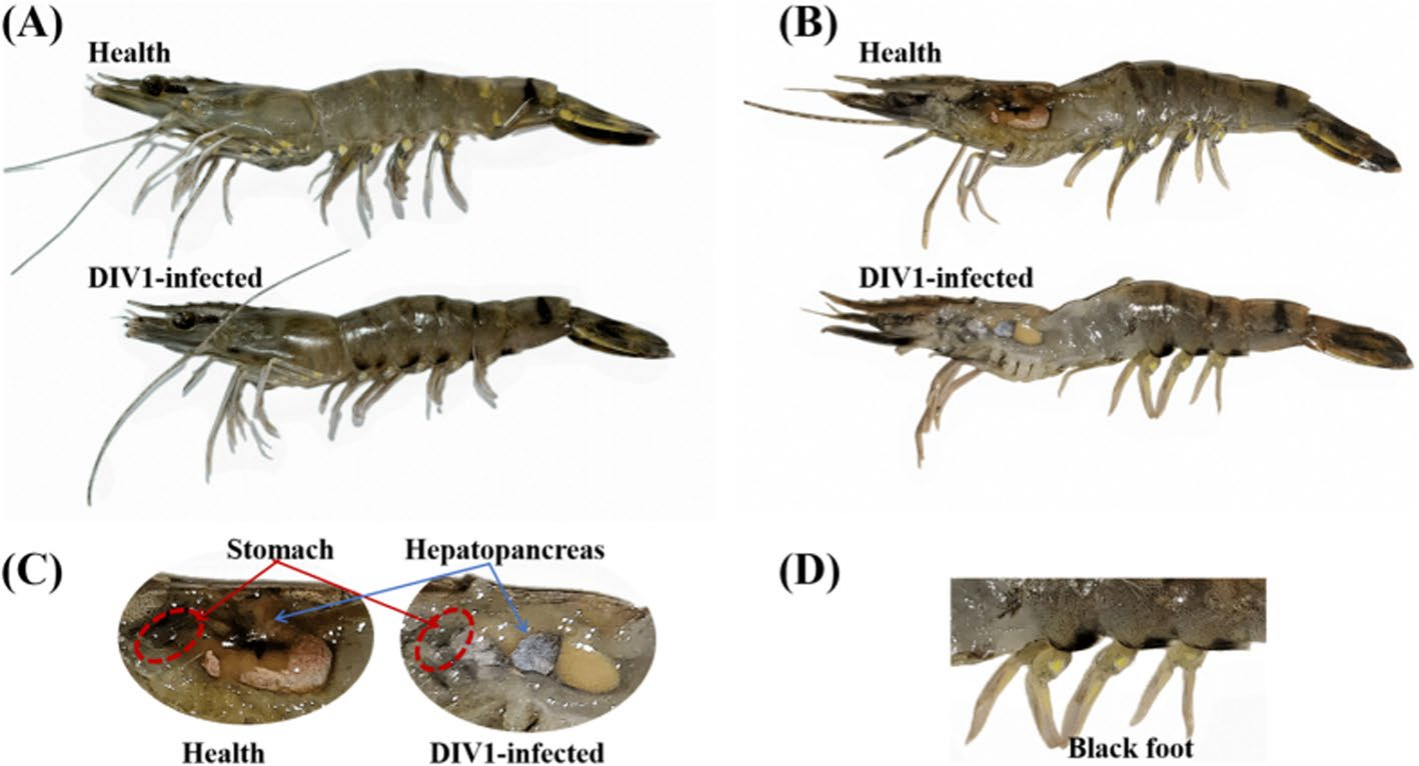
Symptoms of DIV1 infection in *P. monodon*. **A** Overall symptoms. **B** Head and gut anatomy. **C** Gastric and hepatopancreatic symptoms. **D** Black foot

*P. monodon* infected with DIV1 are dark and black (Fig. 3AB), with a soft shell, turbid and atrophied hepatopancreas, empty stomach and intestines, and reduced mobility until death (Fig. 3C). In addition to the reddish ventral peduncle, black precipitation ("black foot") at the base of the ventral peduncle was detected (Fig. 3D).

**Fig. 4:**
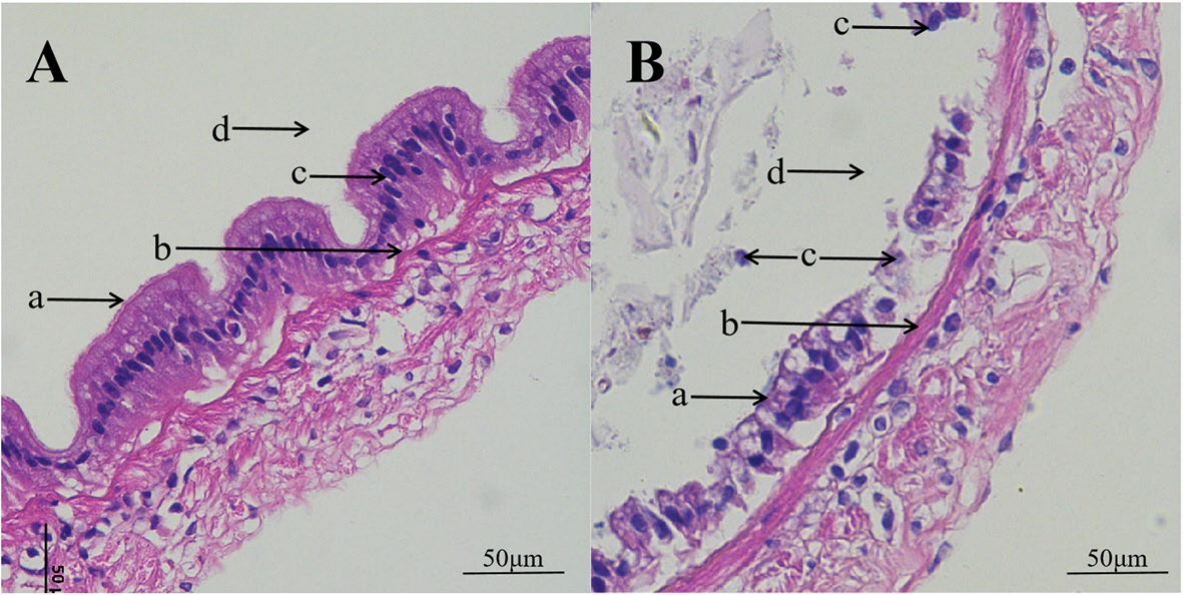
Histopathological analysis of DIV1 of the intestinal mucosa of *P. monodon*. **A** Sections of intestinal tract of healthy *P. monodon.* **B** Sections of intestinal tract of *P. monodon* infected with DIV1; a: brush border; b: epithelial cells; c: nuclei; d: lumen

Sections of *P. monodon* intestine are shown in Fig. 4. Figure 4A is a healthy intestinal section derived from *P. monodon.* Figure 4B represents a section of the intestine infected with DIV1. The intestinal mucosa of *P. monodon* infected with the virus was damaged. The epithelial cells were separated from the basement membrane, deformed and twisted, and the nuclei were scattered in the intestinal lumen (Duan et al. 2021).

### Results of TaqMan qPCR assay

**Fig. 5:**
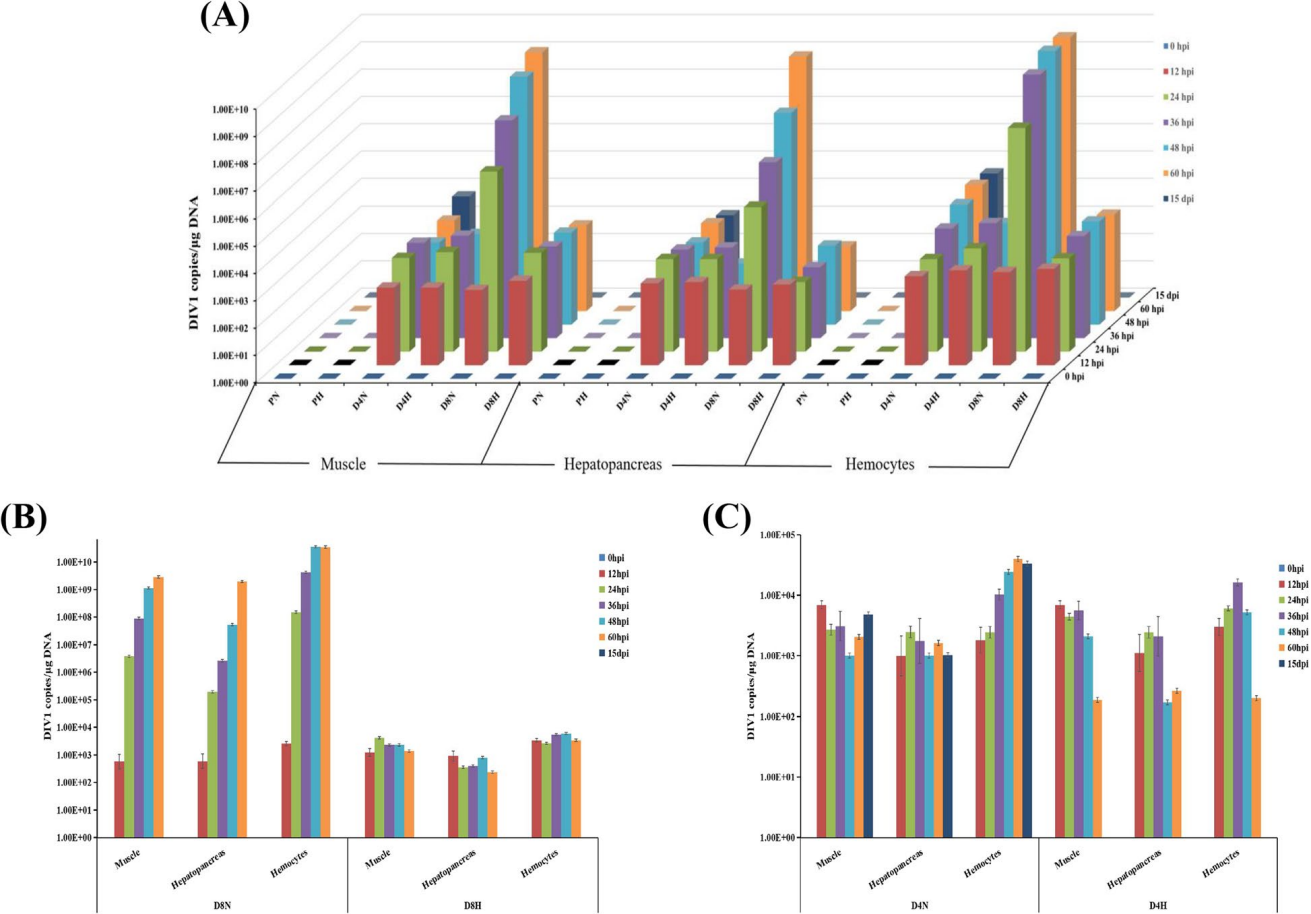
Results of TaqMan probe real-time quantitative PCR assay of *P. monodon*. **A** The x-coordinate represents different tissues in the seven groups. The y-coordinate represents the time point at which the tissues were sampled, and the z-coordinate is the copy number of DIV1. **B** D8N、D8H group results of TaqMan probe real-time quantitative PCR assay of *P. monodon*. **C** D4N、D4H group results of TaqMan probe real-time quantitative PCR assay of *P. monodon*

DNA was extracted from muscle, hepatopancreas and hemolymph of *P. monodon* for qPCR at 0 hpi, 12 hpi, 24 hpi, 36 hpi, 48 hpi, 60 hpi, and 15 dpi of different concentrations of viral solution into the body. The viral copy status and copy rates of DIV1 in *P. monodon* were determined as shown in Fig. 5. The negative control group was found to have 0 copies/μg DNA after sampling at different time points at 26 ± 1 °C (PN) and 34 ± 1 °C (PH). Sampling of 1.12 × 10^4^ copies/μg DNA and 1.12 × 10^8^ copies/μg DNA at different time points yielded zero copies at 26 ± 1 °C (D4N) and 34 ± 1 °C (D4H), respectively. High water temperature (34 ± 1 °C) inhibited viral replication, and the viral copy number was 0 copies/μg DNA at 15 dpi.

### Results of nested PCR assay

**Fig. 6:**
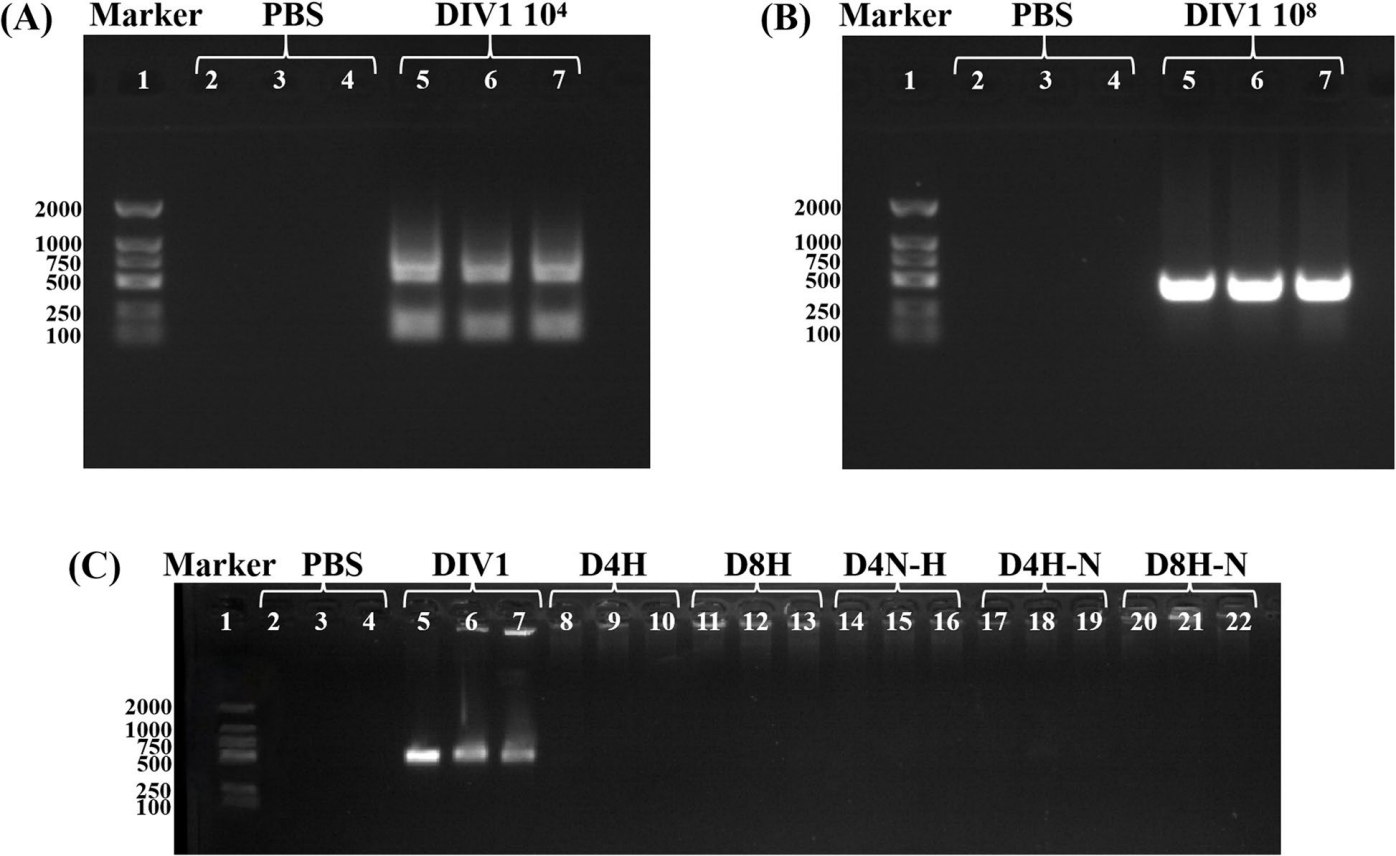
Results of DIV1 detection by nested PCR of blood cells in *P. monodon*. Lane 1 is Marker: DL2000. Lanes 2–4 are PBS negative controls. **A** Lanes 5–7 are DIV1 infected with 3.49 × 10^4^ copies/μg DNA. **B** Lanes 5–7 are DIV1 infected with 2.15 × 10^8^ copies/μg DNA. **C** The lanes are divided into seven groups, with three parallels in each group (left to right): PBS negative control, DIV1 positive control (1.12 × 10^8^ copies/μg DNA), D4H, D8H, D4N-H, D4H-N, and D8H-N

The results of the PCR assay for the detection of DIV1 in blood cells of *P. monodon* by nested PCR are shown in Fig. 6A, B. Using a DL2000 molecular weight marker, lanes were divided into three groups, with three parallels each. Lanes 2–4 in the first group represented the negative control PBS group, and the results of the assay were negative. Lanes 5–7 in the second group represented the D4-infected (3.49 × 10^4^ copies/μg DNA) and the D8-infected (2.15 × 10^8^ copies/μg DNA) groups, and the results of the assay were both positive. The results of DIV1-nested PCR in blood cells of *P. monodon* after high temperature (34 ± 1 °C) and variable temperature tests are shown in Fig. 4C. All lanes are divided into seven groups with three parallel lines in each group. The seven groups were PBS negative control, DIV1 positive control, D4H, D8H, D4N-H, D4H-N, D8H-N, respectively. The muscle tissue of rejuvenated *P. monodon* was selected as the DIV1 positive control group. The virus copy number was 1.12 × 10^8^ copies/μg DNA by qPCR. The remaining five groups were muscle tissue of 15dpi *P. monodon*. The virus copy numbers were all 0 copies/μg DNA by qPCR.

## Discussion

Shrimp aquaculture has always been an important means worldwide to obtain high quality protein. It represents a major economic source in coastal areas. Water temperature is one of the important environmental factors in aquaculture. It has a significant impact on the growth and development of aquatic organisms. Viral diseases are frequent in shrimp aquaculture,. DIV1 is a novel virus with very high virulence and lethality, which seriously affects the development of shrimp aquaculture. A close relationship exists between water temperature and viral infection and transmission. It is important to understand the effect of water temperature on viral infection or the prevention and control of shrimp virus disease. It has been reported that viral infections in *Pro. clarkii*, *L. vannamei*, *Mar. japonicus* and *Mac. Nipponense* are affected by water temperature. Elevated water temperature decreases the rates of viral replication and transmission and attenuates the infectivity of the virus (Du et al. 2008, 2006; Granja et al. 2006; Withyachumnarnkul et al. 2003). The proliferation of white spot syndrome virus (WSSV) was suppressed when the water temperature was 32 °C to 35 °C, which extended the survival time of *Pro. clarkii* compared with water at temperatures of 22 °C to 27 °C (Jiravanichpaisal et al. 2004; Luo, 2010). This suggests that water temperature has a significant effect on the biology of the virus. By feeding *L. vannamei* infected with WSSV, it was found that the mortality rate of *L. vannamei* at 32 °C was lower than at 26 °C, due to significant inhibition of viral replication (Granja et al. 2006). Intramuscular injection of WSSV-infected Vannamei shrimps at 32 °C or 33 °C was effective in suppressing viral replication (Rahman et al. 2007, 2006). Radical thermal therapy, in which the water temperature was increased to 36 °C, led to the eradication of DIV1 infection from the shrimp" (Guo et al. 2022). This indicates that high water temperature can inhibit the survival and transmission of the virus through various ways, and shrimp culture has a positive prevention and control effect. Within 24 to 72 hpi with WSSV, the amount of virus carried by *Mar. japonicus* treated at room temperature was significantly higher than at high temperature. This study confirmed that high water temperature reduced the mortality of WSSV-infected shrimp by inhibiting viral replication (You et al. 2010). The levels of WSSV were lower in Japanese swamp shrimp under high temperature (32 °C), and high water temperature had a specific inhibitory effect on WSSV (Yuan, 2020). In addition to WSSV and DIV1, high water temperature also inhibited the levels of TSV and IHHNV (Montgomery-Brock et al. 2007). This suggests that water temperature has a general regulatory effect on different types of viruses. Water temperature regulated the strength and effectiveness of the immune response in shrimp at moderate temperatures.Elevated water temperature enhanced the immune response of shrimps and improved their resistance to viruses. Elevated water temperature led to a decline in dissolved oxygen in the water. Persistent low-oxygen environment raised the hemocyanin levels of crustaceans. Hemocyanin is an important immune factor in crustaceans, with phenol oxidase activity and antimicrobial function. Elevated temperature can partially increase immunity (Pan et al. 2008; Zhang et al. 2007). Wu Xiaoguo et al. (Wu et al. 2012) reported that shrimp white spot syndrome virus infection induced stress response in host cells under higher culture water temperature, and stimulated a series of host cell defense mechanisms and reduced the mortality. In addition, water temperature has a significant effect on the growth and physiology of shrimps. In general, the influence of water temperature on virus infection in shrimp culture is multi-sided, which not only directly inhibit the survival and transmission of virus, but also enhance the immune ability of shrimp, jointly maintain the health of shrimp population.

While the effect of temperature on DIV1 infection in *P. monodon* has yet to be reported, the present study findings suggest that increasing the temperature of the water body eliminated the infection. Based on a comprehensive analysis of survival curves and LC_50_ values, clinical symptoms, results of nested PCR, qPCR, and histopathological studies, it was found that high water temperature (34 ± 1 °C) inhibited and eliminated DIV1 infection from *P. monodon.* Thus, the results indicate the conditions for preventing the outbreaks of DIV1 and eliminating the virus. The findings provide a basis for the subsequent elucidation of the mechanism of viral inhibition and eradication.

Analyzing the survival curve, we observe a proportional increase in mortality over time in the D8N group of *P. monodon* during the acute infection phase at room temperature (26 ± 1 °C), reaching a 100% mortality rate after 128 hpi. However, in the D4N group with latent infection at room temperature (26 ± 1 °C), despite one unexpected death after 4 hpi, we have excluded this case from statistical consideration, ensuring the reliability of the experimental results. Overall, except for the D8N group, no deaths due to viral infection were observed in other groups, highlighting the acute lethal effects of DIV1 infection on *P. monodon*. DIV1-infected *P. monodon* exhibit a range of clinical symptoms, including red body, soft shell, black foot, hepatopancreas turbidity, and empty intestinal and stomach. These observed symptoms provide an intuitive understanding of the overall health status of *P. monodon* infected with DIV1. Further histopathological analysis reveals the impact of DIV1 infection on the intestines of *P. monodon*. Compared to healthy controls, intestinal sections from the infected group show damage to the intestinal mucosa, separation of epithelial cells from the basal membrane, deformation and twisting of cell nuclei, with nuclei scattered in the gut lumen. These pathological changes highlight the destructive effects of DIV1 on the intestinal tissue structure and cell morphology of *P. monodon*.

The qPCR results following DIV1 injection at 0, 12, 24, 36, 48, 60 hpi, and 15 dpi indicate viral copy numbers in the muscle, hepatopancreas, and hemolymph of *P. monodon*. Notably, post-infection, the viral replication rate is higher at 26 ± 1 °C compared to 34 ± 1 °C, with the fastest replication occurring between 12 and 24 hpi, particularly in the hemolymph. Beyond 48 hpi under elevated temperature conditions, there is a declining trend in viral copy numbers inversely proportional to time, culminating in complete viral elimination by 15 dpi.

In conjunction with the PCR results, the successful establishment of an artificial injection model for both latent infection (D4H) and acute infection (D8H) DIV1 infections is evident. Following 15 days of high-temperature cultivation, detection results for *P. monodon* in both latent and acute infection groups show negative readings, signifying the complete elimination of the virus. Additionally, the outcome of the temperature shift experiment, where prawns were transferred to room temperature (26 ± 1 °C) after 15 days of high-temperature cultivation, indicates no viral resurgence, with all detection results displaying negativity. This phenomenon underscores the thorough eradication of the virus and the absence of any potential for recurrence.

In conclusion, the findings presented in this study hold significant implications for the field of aquaculture, particularly in combating DIV1 infections in *P. monodon*. The observed temperature-dependent dynamics of viral replication provide valuable insights into the environmental factors influencing viral pathogenesis. The successful establishment of artificial infection models and the subsequent complete elimination of the virus, even upon temperature shifts, underscore the robustness and efficacy of high-temperature treatments. This not only contributes to our understanding of DIV1 biology but also opens avenues for developing practical strategies to prevent and control viral outbreaks in aquaculture. Looking forward, this research sets the stage for further exploration of the underlying mechanisms driving the observed temperature-dependent responses in viral infections. Future studies could delve into the molecular pathways involved, shedding light on how elevated temperatures affect viral replication and the host immune response. Additionally, the application of high-temperature treatments as a preventive measure holds promise for implementation in aquaculture practices. Further investigations into optimizing temperature-based interventions, considering long-term effects and sustainability, will be crucial for developing holistic and effective approaches to manage viral diseases in shrimp farming. Overall, this study not only addresses a critical issue in aquaculture but also lays the groundwork for future advancements in the prevention and control of viral infections, contributing to the sustainable development of shrimp farming industries worldwide.

## Conclusions

In this study, we found that DIV1 in *P. monodon* was completely eliminated by increasing the culture water temperature to 34 ± 1 °C. No recurrence of DIV1was detected after 7 days of culture at 34 ± 1 °C and transfer to water at 26 ± 1 °C.

## Data Availability

The data that support the findings of this study are available on request from the correspondingauthor. The data are not publicly available due to privacy or ethical.
